# Supplementary material for: Unveiling the Bioactive Potential of the Invasive Jellyfish Phyllorhiza punctata Through Integrative Transcriptomic and Proteomic Analyses
Source: Biomolecules. 2025 Aug 4;15(8):1121. doi: 10.3390/biom15081121 (PMC12383608; doi:10.3390/biom15081121)
Supplement: Supplementary file 1 [file biomolecules-15-01121-s001.zip › Suplementary Material/FileS3/PEPFOLD4/XP_065054022_5269/clusters.html]

 PEP-FOLD clusters


Download archive of all models  here   

|  |  |  |
| --- | --- | --- |
| # | file | sOPEP |
| # cluster 1 - Size: 22 |  |  |
|  | XP\_065054022\_5269-model1 | -11.663 |
|  | XP\_065054022\_5269-model1.1 | -11.663 |
|  | XP\_065054022\_5269-model1.2 | -11.663 |
|  | XP\_065054022\_5269-model1.3 | -11.663 |
|  | XP\_065054022\_5269-model1.4 | -11.663 |
|  | XP\_065054022\_5269-model1.5 | -11.663 |
|  | XP\_065054022\_5269-model1.6 | -11.663 |
|  | XP\_065054022\_5269-model1.7 | -11.663 |
|  | XP\_065054022\_5269-model1.8 | -11.663 |
|  | XP\_065054022\_5269-model1.9 | -11.663 |
|  | XP\_065054022\_5269-model1.10 | -11.663 |
|  | XP\_065054022\_5269-model1.11 | -11.663 |
|  | XP\_065054022\_5269-model1.12 | -11.663 |
|  | XP\_065054022\_5269-model1.13 | -11.663 |
|  | XP\_065054022\_5269-model1.14 | -11.663 |
|  | XP\_065054022\_5269-model1.15 | -11.663 |
|  | XP\_065054022\_5269-model1.16 | -11.663 |
|  | XP\_065054022\_5269-model1.17 | -11.663 |
|  | XP\_065054022\_5269-model1.18 | -11.663 |
|  | XP\_065054022\_5269-model1.19 | -11.663 |
|  | XP\_065054022\_5269-model1.20 | -11.5652 |
|  | XP\_065054022\_5269-model1.21 | -11.5652 |
| # cluster 2 - Size: 14 |  |  |
|  | XP\_065054022\_5269-model2 | -5.82595 |
|  | XP\_065054022\_5269-model2.1 | -5.78112 |
|  | XP\_065054022\_5269-model2.2 | -5.78112 |
|  | XP\_065054022\_5269-model2.3 | -5.70413 |
|  | XP\_065054022\_5269-model2.4 | -5.60147 |
|  | XP\_065054022\_5269-model2.5 | -5.06824 |
|  | XP\_065054022\_5269-model2.6 | -5.06824 |
|  | XP\_065054022\_5269-model2.7 | -4.96285 |
|  | XP\_065054022\_5269-model2.8 | -4.90222 |
|  | XP\_065054022\_5269-model2.9 | -4.62555 |
|  | XP\_065054022\_5269-model2.10 | -3.94759 |
|  | XP\_065054022\_5269-model2.11 | -3.35439 |
|  | XP\_065054022\_5269-model2.12 | -3.12338 |
|  | XP\_065054022\_5269-model2.13 | -3.04438 |
| # cluster 3 - Size: 18 |  |  |
|  | XP\_065054022\_5269-model3 | -5.73208 |
|  | XP\_065054022\_5269-model3.1 | -4.83031 |
|  | XP\_065054022\_5269-model3.2 | -4.83031 |
|  | XP\_065054022\_5269-model3.3 | -4.80049 |
|  | XP\_065054022\_5269-model3.4 | -4.3485 |
|  | XP\_065054022\_5269-model3.5 | -4.072 |
|  | XP\_065054022\_5269-model3.6 | -4.06323 |
|  | XP\_065054022\_5269-model3.7 | -4.03148 |
|  | XP\_065054022\_5269-model3.8 | -3.98155 |
|  | XP\_065054022\_5269-model3.9 | -3.84669 |
|  | XP\_065054022\_5269-model3.10 | -3.77743 |
|  | XP\_065054022\_5269-model3.11 | -3.74973 |
|  | XP\_065054022\_5269-model3.12 | -3.65391 |
|  | XP\_065054022\_5269-model3.13 | -3.5104 |
|  | XP\_065054022\_5269-model3.14 | -3.40953 |
|  | XP\_065054022\_5269-model3.15 | -3.37612 |
|  | XP\_065054022\_5269-model3.16 | -2.93892 |
|  | XP\_065054022\_5269-model3.17 | -2.91252 |
| # cluster 4 - Size: 18 |  |  |
|  | XP\_065054022\_5269-model4 | -5.22169 |
|  | XP\_065054022\_5269-model4.1 | -5.1166 |
|  | XP\_065054022\_5269-model4.2 | -5.08695 |
|  | XP\_065054022\_5269-model4.3 | -5.047 |
|  | XP\_065054022\_5269-model4.4 | -5.04518 |
|  | XP\_065054022\_5269-model4.5 | -5.03122 |
|  | XP\_065054022\_5269-model4.6 | -5.03122 |
|  | XP\_065054022\_5269-model4.7 | -4.94924 |
|  | XP\_065054022\_5269-model4.8 | -4.92564 |
|  | XP\_065054022\_5269-model4.9 | -4.89126 |
|  | XP\_065054022\_5269-model4.10 | -4.88371 |
|  | XP\_065054022\_5269-model4.11 | -4.87197 |
|  | XP\_065054022\_5269-model4.12 | -4.64944 |
|  | XP\_065054022\_5269-model4.13 | -4.59921 |
|  | XP\_065054022\_5269-model4.14 | -4.5118 |
|  | XP\_065054022\_5269-model4.15 | -4.48862 |
|  | XP\_065054022\_5269-model4.16 | -4.35348 |
|  | XP\_065054022\_5269-model4.17 | -4.03302 |
| # cluster 5 - Size: 1 |  |  |
|  | XP\_065054022\_5269-model5 | -3.85188 |
| # cluster 6 - Size: 9 |  |  |
|  | XP\_065054022\_5269-model6 | -3.61906 |
|  | XP\_065054022\_5269-model6.1 | -3.61276 |
|  | XP\_065054022\_5269-model6.2 | -3.52001 |
|  | XP\_065054022\_5269-model6.3 | -3.46692 |
|  | XP\_065054022\_5269-model6.4 | -3.4267 |
|  | XP\_065054022\_5269-model6.5 | -3.3817 |
|  | XP\_065054022\_5269-model6.6 | -3.28633 |
|  | XP\_065054022\_5269-model6.7 | -3.06586 |
|  | XP\_065054022\_5269-model6.8 | -2.88164 |
| # cluster 7 - Size: 2 |  |  |
|  | XP\_065054022\_5269-model7 | -3.0919 |
|  | XP\_065054022\_5269-model7.1 | -3.03562 |
| # cluster 8 - Size: 1 |  |  |
|  | XP\_065054022\_5269-model8 | -2.98345 |
| # cluster 9 - Size: 4 |  |  |
|  | XP\_065054022\_5269-model9 | -2.90233 |
|  | XP\_065054022\_5269-model9.1 | -2.89207 |
|  | XP\_065054022\_5269-model9.2 | -2.87061 |
|  | XP\_065054022\_5269-model9.3 | -2.8014 |
| # cluster 10 - Size: 1 |  |  |
|  | XP\_065054022\_5269-model10 | -2.77496 |
| # cluster 11 - Size: 2 |  |  |
|  | XP\_065054022\_5269-model11 | -2.6016 |
|  | XP\_065054022\_5269-model11.1 | -2.53507 |
| # cluster 12 - Size: 2 |  |  |
|  | XP\_065054022\_5269-model12 | -2.57777 |
|  | XP\_065054022\_5269-model12.1 | -2.52995 |
| # cluster 13 - Size: 6 |  |  |
|  | XP\_065054022\_5269-model13 | -2.56522 |
|  | XP\_065054022\_5269-model13.1 | -2.55041 |
|  | XP\_065054022\_5269-model13.2 | -2.55 |
|  | XP\_065054022\_5269-model13.3 | -2.54778 |
|  | XP\_065054022\_5269-model13.4 | -2.54713 |
|  | XP\_065054022\_5269-model13.5 | -2.54713 |
|  |  |  |
